# Adherence to vitamin and dietary supplement intake in fertility and pregnancy care: insights into knowledge, information satisfaction, and formulation variability

**DOI:** 10.1007/s00404-025-08288-w

**Published:** 2026-01-07

**Authors:** Nele-Juliana Breuste, Cordula Schippert, Frauke von Versen-Höynck

**Affiliations:** Department of Obstetrics and Gynecology, Hannover Medical School, Carl-Neuberg-Straße 1, 30625 Hannover, Lower Saxony Germany

**Keywords:** Patient education, Micronutrients, Supplementation, Behavior

## Abstract

**Purpose:**

This study investigated adherence to vitamin and dietary supplement intake, satisfaction with healthcare-provided information, and knowledge of essential micronutrients among women seeking fertility treatment and pregnant women in Germany.

**Methods:**

An anonymous online survey (34 questions) assessed sociodemographics, supplement intake, knowledge and motivations. Adherence and satisfaction were measured by MARS-D (Medication Adherence Rating Scale) and SIMS-D (Satisfaction with Information about Medicines Scale).

**Results:**

Among 254 participants, 93.7% reported supplement use, and 86.6% began intake preconceptionally. On average, participants consumed two (2.0 ± 1.36) supplements concurrently. Most multiple micronutrient supplements (MMS) contained folic acid (100%) and iodine (86.2%) at recommended doses, other nutrients varied considerably. Participants knew two (1.81 ± 1.43) out of six micronutrients prior to information provision, increasing to three (2.94 ± 1.65) afterwards. Satisfaction with information (SIMS-D: 7.46 ± 5.92) was low, whereas adherence was high (MARS-D: 27.16 ± 3.06). Higher information satisfaction was associated with pregnancy (*p* = 0.007), younger age (*p* = 0.009), and lower educational level (*p* = 0.024). Adherence was linked to trimester (*p* = 0.007) and region (*p* = 0.013), with higher MARS-D scores in the first trimester and among participants from North Rhine-Westphalia. Key motivations were protecting the child and preventing deficiencies; main barriers included lack of awareness and feeling overwhelmed by preparation oversupply.

**Conclusions:**

Despite high adherence, knowledge and satisfaction with information remain limited. The wide variability in MMS formulations may pose risks of over- or underdosage. Combining personalized consultations with trustworthy media resources is essential to assess individual needs and provide detailed recommendations.

**Supplementary Information:**

The online version contains supplementary material available at 10.1007/s00404-025-08288-w.

## What does this study add to the clinical work

**Table:** 

This study shows high supplement adherence in fertility and pregnancy care, but highlights gaps in detailed knowledge, information satisfaction, and therefore in education, and an inconsistency in multiple micronutrient supplement formulations. It emphasizes the need for personalized counseling and improved, targeted educational strategies to optimize supplement use and prevent both under- and overdosage.	

## Introduction

Micronutrient requirements increase preconceptionally and prenatally [1, 2]. The German Nutrition Society (DGE) recommends daily supplementation of 400 µg of folic acid starting at least four weeks before conception to prevent congenital abnormalities such as neural tube defects, cardiac defects or anemia [3–9]. Additionally, supplements should contain 100–150 µg of iodine, due to its relevance for maternal thyroid function and fetal neurodevelopment [7, 9–12]. However, iodine supplementation is contraindicated in hyperthyroidism and requires individual physician oversight [9]. Further supplementation is only recommended by the DGE in case of specific deficiencies [7]. Iron deficiency may lead to anemia, intrauterine growth retardation and neonates small for gestational age [6, 13, 14]. The daily intake is set for 30 mg [7]. DHA, which is important for fetal neurological development and the prevention of preterm birth, should be supplemented at a dose of 200 mg per day if dietary intake of fatty sea fish or algae products is insufficient [7, 15]. Vitamin B12 supplementation is important for individuals following vegetarian or vegan diets who might be unable to meet the daily intake of 4.5 µg per day [7, 16]. Deficiency is associated with neural tube defects, megaloblastic anemia, neurological and development complications, and an increased risk of miscarriage [4, 6, 16]. Low vitamin D levels may increase the risk of preeclampsia, gestational diabetes, low birth weight and developing infantile asthma [17–21]. An additional daily intake of 800 I.U. (20 µg) is recommended, particularly for individuals with limited sun exposure [18]. The European Food Safety Authority (EFSA) recommends a daily choline intake of 480 mg during pregnancy, as inadequate intake may increase the risk of neural tube defects, and a sufficient intake contributes to fetal liver function [22–24].

To ensure adequate intake, a recommendation should ideally be provided preconceptionally by the gynecologist. Nevertheless, previous studies have shown low adherence, especially preconceptionally [25–29]. Both under- and overdosages have been reported [25, 27].

This study aimed to expand current insights on vitamin and dietary supplement intake among women seeking fertility treatment and pregnant women in Germany. It evaluated adherence and satisfaction with healthcare-provided information, the effect of information on participants’ awareness of recommended micronutrients and the role of different information sources. The composition of selected multiple micronutrient supplements (MMS) was analyzed. Participants’ motivations and barriers to supplement intake were explored.

## Methods

### Participants

Women seeking fertility treatment and pregnant women were invited to participate in an anonymous online questionnaire. The study was approved by the Clinical Ethics Committee of Hannover Medical School (May 16, 2024; No. 11415_BO_K_2024). Participants were recruited through flyers and posters in gynecological and fertility clinics in the federal state of Lower Saxony, and online via social media and pregnancy-related websites. Eligible were individuals aged 18 or older who were either pregnant or undergoing fertility treatment. Ineligible responses were excluded from the dataset.

### Questionnaire

The cross-sectional, anonymous online questionnaire was created in German using SoSci-Survey (Version 3.6.09). Following pre-test revisions, the questionnaire was made available from July 26th, 2024, to January 26th, 2025.

The questionnaire contained a total of 34 questions on sociodemographic parameters, supplement intake, adherence, information satisfaction, knowledge and motivations. The individual number of questions varied based on the participants’ characteristics through filtered questions. An English version of the questionnaire can be found in the supplementary material. Knowledge was assessed by asking how many of six micronutrients recommended by DGE and EFSA [7, 23] were known before and after receiving information. Participants’ subjective satisfaction with the received medical information was assessed using the SIMS-D, the German version of the “Satisfaction with Information about Medicines Scale”, including items about medication use and risks. The SIMS-D scores only “about right” and “none needed” as one point, other responses (“too much”, “too little”, “no information received”) score zero. Higher scores indicate greater satisfaction [30, 31]. At the end of the original questionnaire, participants were asked to assess information regarding correct dose and choice of preparation. Adherence was assessed using the MARS-D, the German version of the “Medication Adherence Rating Scale”, which evaluates self-reported adherence on a 5-point Likert scale (“always” = 1 to “never” = 5), with higher scores reflecting higher adherence [32, 33]. We extended the questionnaire with an item assessing intake exceeding recommended dosages. Usage permission for both questionnaires was granted by the originator.

### Data analysis

Only fully completed questionnaires were considered to ensure a comprehensive data set for analysis. Descriptive statistics were calculated for each question. SIMS-D and MARS-D scores were analyzed overall, by subgroups, and within subscales. Paired *t*-tests and calculations of Cohen’s *d* were performed to compare the mean number of known micronutrients out of six recommended ones before and after receiving information. Frequencies of awareness of each micronutrient were also determined pre- and post-information.

The number of concurrently used MMS was assessed. MMS used by at least two participants were evaluated regarding the inclusion of folic acid, iodine, DHA, iron, vitamin B12, vitamin D and choline and doses were analyzed using descriptive statistics, alongside an assessment of the presence of additional substances. Single-ingredient supplement use was assessed regarding frequency of consumption and dose range using descriptive statistics.

Correlations with SIMS-D and MARS-D scores were calculated using variable-appropriate methods. For continuous variables, Pearson’s *r* was used. For ordinal variables, Spearman’s *ρ* was applied. Categorical variables were analyzed using Eta-squared and univariate ANOVA. Chi-Square tests assessed the correlation between primary source of information and the start of supplementation. A *p*-value of < 0.05 was considered statistically significant, though all correlations and significances should be interpreted as exploratory. Motivational aspects were examined by calculating frequencies, means and standard deviation (SD) for each statement. Statements with mean scores < 2 (Likert scale: 1 = “totally disagree”; 2 = “disagree”) were considered not relevant to the participants. Data analysis was performed using IBM SPSS Statistics Version 30 (IBM Corp., Armonk, NY, USA).

## Results

### Participants and characteristics

Out of 957 link clicks, 372 participants initiated the survey (response rate: 38.9%), and 254 valid cases were completed (completion rate: 68.3%). The average completion time was 14.27 ± 1.4 min.

Sociodemographic characteristics are presented in Table 1. Of the participants, 103 (40.6%) were undergoing fertility treatment, whereas 151 (59.4%) were already pregnant. The majority were aged 30–39 years (173; 68.1%), highly educated (tertiary education completed: 176; 69.2%), employed full-time (133; 52.4%), and primarily living in Lower Saxony (164; 64.6%) or North Rhine-Westphalia (64; 25.2%).

**Table 1:** Sociodemographic information of participants

Sociodemographic information		
Characteristics	Participants	%
General information		
Age (age distribution ranged exclusively between 21 and 49 years)	*n* = 254	100.0
21–29	64	25.2
30–39	173	68.1
40–49	17	6.7
Highest level of education completed	*n* = 254	100.0
Completion of secondary school (9th grade, “Hauptschulabschluss”)	2	0.8
Completion of intermediate school (10th grade, “Realschulabschluss”)	8	3.1
Vocational Higher Education Entrance Qualification (“Fachabitur”)	6	2.4
Completion of highschool (general university entrance qualification, “Abitur”)	16	6.3
Completed vocational training	46	18.1
University of applied sciences degree (“Fachhochschulabschluss”)	26	10.2
University degree	122	48.0
Higher academic qualification (doctorate/habilitation)	28	11.0
Status of employment	*n* = 254	100.0
Employed full-time	133	52.4
Employed part-time	62	24.4
Seeking work	1	0.4
Independently	5	2.0
On parental leave	22	8.7
On leave/unable to work	7	2.8
At school/university/training	19	7.5
None of the above	5	2.0
Federal state of residence	*n* = 254	100.0
Lower Saxony	164	64.6
North Rhine-Westphalia	64	25.2
Others or not currently living in Germany (a detailed list of all responses is provided in *Supplemental *Table 1)	26	10.2

Reproductive Information		
Reproductive status	*n* = 254	100.0
Seeking fertility treatment	103	40.6
Pregnant	151	59.4
Current trimester	*n* = 151	100.0
First	28	18.5
Second	47	31.1
Third	76	50.3
Previous pregnancies	*n* = 254	100.0
No	115	45.3
Yes	139	54.7
Number of previous pregnancies	*n* = 139	100.0
1	66	47.5
2	48	34.5
3	15	10.8
4	3	2.2
5	4	2.9
6	3	2.2
Number of live births	*n* = 139	100.0
0	38	27.3
1	75	54.0
2	20	14.4
3	5	3.6
4	1	0.7
Number of miscarriages	*n* = 139	100.0
0	74	53.2
1	50	36.0
2	11	7.9
3	2	1.4
5	2	1.4
Number of abortions	*n* = 139	100.0
0	121	87.1
1	17	12.2
2	1	0.7
Number of ectopic pregnancies	*n* = 139	100.0
0	131	94.2
1	7	5.0
2	1	0.7
Number of fertility treatments	*n* = 254	100.0
0	113	44.5
1	99	39.0
2	30	11.8
3	7	2.8
4	4	1.6
5	1	0.4
Type of treatment	*n* = 200	100.0
Timed intercourse	112	56.0
Intrauterine insemination	16	8.0
IVF /ICSI	44	22.0
Frozen embryo transfer cycle	17	8.5
Others	11	5.5

ICSI = intracytoplasmic sperm injection; IVF = in vitro fertilization

### Vitamin and dietary supplement intake

Information on supplement intake is presented in Table 2. A total of 238 (93.7%) participants reported current intake of vitamins or dietary supplements, with 206 (86.6%) having started supplementation preconceptionally. Among those currently taking supplements, 104 (43.7%) were women seeking fertility treatment and 134 (56.3%) were already pregnant. Overall, participants consumed a mean of two (2.0 ± 1.36) preparations simultaneously.

**Table 2:** Participants’ information on dietary supplementation

Information on dietary supplementation		
Primary source of information	*n* = 254	100.0
Gynecologist	96	37.8
Internet	86	33.9
Others*	70	27.6
I did not receive any information	2	0.8
Current Intake of dietary supplements	*n* = 254	100.0
No	16	6.3
Yes	238	93.7
Start of Intake	*n* = 238	100.0
Preconceptional	206	86.6
Postconceptional Since the first trimester Since the second trimester	32 30 2	13.4 12.6 0.8
Intake planned	*n* = 16	100.0
Yes	9	56.3
No	2	12.5
I am undecided	5	31.3
If no intake is planned how much pressure do you feel regarding the intake of vitamins and dietary supplements for conception or during pregnancy?	*n* = 2	100.0
4 = strong	2	100.0
Previous supplement intake aside from pregnancy and conception	*n* = 254	100.0
Yes	159	62.5
No	95	37.4
Experiences of complications during conception or pregnancy in the social environment, which were likely associated with a perceived lack of dietary supplementation	*n* = 254	100.0
Yes	21	8.3
No	233	91.7

^*^a detailed list of all responses is provided in *Supplemental *Table 2

All 29 multiple micronutrient supplements (MMS) used by at least two participants contained folic acid (100%). Vitamin B12 was included in 27 (93.1%) of the preparations, both iodine and vitamin D in 24 (82.8%), and iron in 18 (62.1%). DHA was present in 12 (41.4%) of the formulations, and choline in seven (24.1%) (overview: *Supplemental *Table 3). Additionally, 24 (82.8%) of the MMS contained up to 22 other substances in varying doses and various combinations. Participants also reported taking single-ingredient supplements: Vitamin D was consumed by 59 (23.2%) participants, and 34 (13.4%) participants reported using iron. 25 (10.5%) participants took single-ingredient supplements of folic acid, while DHA was taken by 19 (7.5%) participants and 18 (7.1%) participants took vitamin B12. Eight (3.4%) participants consumed iodine, and six (2.4%) participants reported using choline (overview: *Supplemental *Table 4). Table 3 shows the consumed dose ranges and recommended intakes for each micronutrient.

**Table 3:** Descriptive statistics for doses of recommended micronutrient. Participant-reported doses for single-ingredient supplements and doses from MMS (consumed by at least two participants, *n* = 29) were analyzed

Micronutrient	Recommended dose per day^a^	Single-ingredient supplements				MMS			
		Min	Max	Mean	SD	Min	Max	Mean	SD
Folic acid (µg)	Additional intake: 400 µg	150	5000	652.08	947.02	400.0	1000	589.66	189.63
Iodine (µg)	Additional intake: 100–150 µg	50	250	156.25	62.32	100.0	150	145.83	14.12
DHA (mg)	Additional intake in case of deficiency: 200 mg	90	1700	578.00	441.90	200.0	300	237.50	43.30
Iron (mg)	Adequate Intake: 30 mg	10	200	52.29	46.86	5.0	454	36.39	104.32
Vitamin B12 (µg)	Adequate Intake: 4.5 µg	1	1000	332.36	334.70	2.6	60	7.90	10.88
Vitamin D (I.U.)	Additional intake in case of deficiency: 800 I.U	50	20,000	3296.80	4769.52	200.0	2000	725.00	362.66
Choline (mg)	Adequate Intake: 480 mg	240	600	356.00	160.09	100.0	130	121.43	14.64

^a^ recommended by the German Nutrition Society (DGE) and the European Food Safety Authority (EFSA)

DHA = Docosahexaenoic acid; MMS = multiple micronutrient supplements

**Table 4:** Knowledge on six recommended micronutrients prior to and after receiving information (A) and impact of information on micronutrient knowledge (B)

(A) Knowledge on six recommended micronutrients					
	*n*	Minimum	Maximum	Mean	SD
Number of known micronutrients pre receiving information	254	0	6	1.81	1.43
Folic acid Iodine Iron Vitamin B12 Vitamin D Choline	228 (89.9%) 64 (25.2%) 62 (24.4%) 49 (19.3%) 54 (21.3%) 4 (1.6%)				
Number of known micronutrients post receiving information	254	0	6	2.94	1.65
Folic Acid Iodine Iron Vitamin B12 Vitamin D Choline	170 (66.9%) 130 (51.2%) 134 (52.8%) 130 (51.2%) 142 (55.9%) 40 (15.7%)				

(B) Impact of information on micronutrient knowledge^a^								
Mean		SD	SEM	95% CI	*t*	df	*p*-value	Cohen’s *d*
Total								
−1.12		1.99	0.13	−1.37; −0.88	−8.998	253	< 0.001	−0.565
Primary source of information: gynecologist								
−0.91		1.82	0.19	−1.26; −0.54	−4.883	95	< 0.001	−0.498
Primary source of information: internet								
−1.30		2.21	0.24	−1.78; −0.83	−5.458	85	< 0.001	−0.589

^a^ paired *t*-test results

### Knowledge and education

**Fig. 1:**
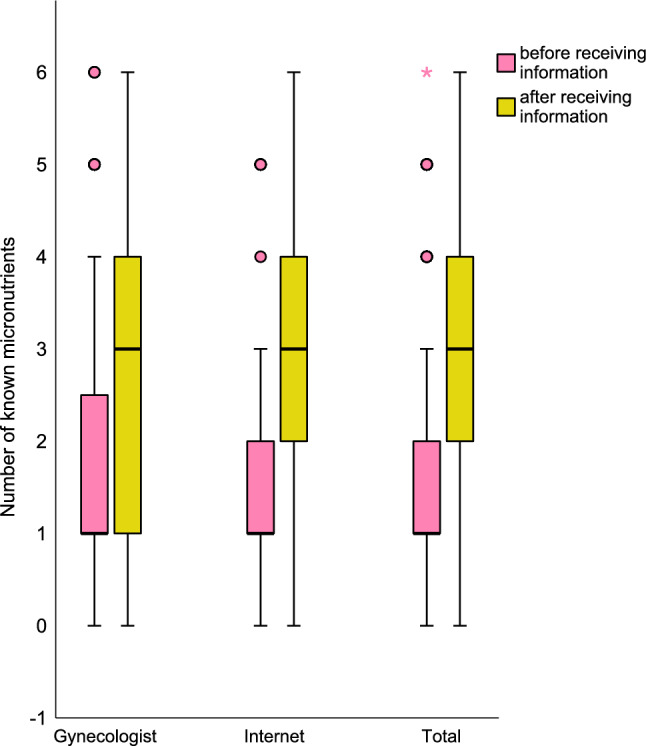
Total number of known micronutrients before and after receiving information, as well as categorized by reproductive status. Dots (◯) represent mild outliers (1.5 – 3 × IQR) and stars (★) represent extreme outliers (> 3 × IQR)

The primary information sources were gynecologists (96; 37.8%) and the internet (86; 33.9%). Participants demonstrated a significant improvement in their knowledge of micronutrients after receiving information (pre-information: 1.81 ± 1.43; post-information: 2.94 ± 1.65; mean difference = -1.12 ± 1.99; *t*(253) = 8.998; *p* < 0.001). The increase was more pronounced among participants who obtained their information from the internet (mean difference = -1.3 ± 2.2; *t*(85) = -5.458), compared to those who consulted a gynecologist (mean difference = -0.91 ± 1.82; *t*(95) = -4.883), but significant in both cases (*p* < 0.001). Effect size calculations using Cohen’s *d* also indicated a small effect for information obtained from gynecologists (*d* = -0.498) and a medium effect for information sourced from the internet (*d* = -0.589). A detailed breakdown of the micronutrient awareness is presented in Table 4, while Fig. 1 illustrates the number of known micronutrients before and after information provision.

The primary source of information did not show a significant correlation to the start of intake of dietary supplements (*p* = 0.097).

### SIMS-D and MARS-D

All scores are presented in Table 5, the corresponding correlations are shown in Table 6. Graphical illustrations of the score results can be found in *Supplemental *Fig. 1 (SIMS-D) and *2* (MARS-D). The overall mean SIMS-D score was 7.46 ± 5.92. SIMS-D scores were significantly correlated with reproductive status (*p* = 0.007), age (*p* = 0.009), and education level (*p* = 0.024). Pregnant participants scored higher (8.28 ± 6.23) than those seeking fertility treatment (6.26 ± 5.24). The mean score for participants aged 21–29 was 8.58 ± 5.68, compared to 5.18 ± 6.85 for those aged 40–49. Those who finished secondary school (“Hauptschule”, 9th grade) achieved a mean score of 13.5 ± 4.95, while participants with a university degree scored 6.4 ± 5.34, and participants with a doctorate or habilitation obtained a mean score of 9.75 ± 7.09.

**Table 5:** SIMS-D and MARS-D scores sorted by reproductive status, along with the variables that demonstrated a statistically significant correlation to the respective scores (as shown in Table 6). Additionally, subscale scores were calculated

Score results					
	*n*	Minimum	Maximum	Mean	SD
SIMS-D					
Total	254	0	19	7.46	5.92
Pregnant	151	0	19	8.28	6.23
Seeking fertility treatment	103	0	19	6.26	5.24
Age 21–29	64	0	19	8.58	5.68
Age 30–39	173	0	19	7.27	5.86
Age 40–49	17	0	19	5.18	6.85
Completion of secondary school (9th grade, “Hauptschulabschluss”)	2	10	17	13.5	4.95
Completion of intermediate school (10th grade, “Realschulabschluss”)	8	2	19	8.00	5.56
Vocational Higher Education Entrance Qualification (“Fachabitur”)	6	3	19	8.83	6.37
Completion of high school (general university entrance qualification, “Abitur”)	16	0	18	8.50	5.07
Completed vocational training	46	0	19	7.87	6.75
University of applied sciences degree (“Fachhochschule”)	26	0	19	7.65	5.62
University degree	122	0	19	6.40	5.34
Higher academic qualification (doctorate/habilitation)	28	0	19	9.75	7.09
MARS-D					
Total	254	12	30	27.16	3.06
Pregnant	149	14	30	27.47	2.77
Seeking fertility treatment	100	12	30	26.71	3.41
First trimester	28	24	30	28.79	1.50
Second trimester	47	14	30	27.21	3.13
Third trimester	74	18	30	27.14	2.77
Federal state of residence:					
Lower Saxony	160	14	30	26.96	3.07
North Rhine-Westphalia	63	21	30	27.98	2.17
others	26	12	30	26.46	4.31
Primary source of information: Gynecologist	94	18	30	27.49	2.66
Primary source of information: internet	84	17	30	27.17	2.82

SIMS-D subscales					
Information in action and usage of medication (question 1–9)	254	0	9	4.28	3.05
Information on potential problems of medication (question 10–17)	254	0	8	2.22	2.86
Information on correct dose of medication (question 18)	254	0	1	0.57	0.50
Information on choice of preparation (question 19)	254	0	1	0.39	0.49

MARS-D subscales regarding variation of doses					
I take less than prescribed	251	1	5	4.71	0.66
I take more than prescribed	252	1	5	4.69	0.75

MARS-D = German Medication Adherence Report Scale; SIMS-D = German Satisfaction with Information about Medicines Scale

**Table 6:** Correlation coefficients between variables and significance of the relationships based on the appropriate correlation method for each variable type

Correlation coefficients			
	Pearson’s *r*		*p*-value
Number of fertility treatments			
SIMS-D score	−0.047		0.454
MARS-D score	−0.022		0.728

	Spearman’s *ρ*		*p*-value
Age			
SIMS-D score	**−0.165**		**0.009**
MARS-D score	−0.073		0.251
Trimester			
SIMS-D score	0.006		0.939
MARS-D score	**−0.222**		**0.007**
Highest level of education completed			
SIMS-D score	**−0.142**		**0.024**
MARS-D score	0.032		0.614
Start of intake			
SIMS-D score	0.011		0.865
MARS-D score	−0.031		0.640

	Chi^2^		*p*-value
Primary source of information and Start of intake	21.199		0.097

	Eta	Eta^2^	*p*-value
Reproductive status			
SIMS-D score MARS-D score	0.168 0.122	0.028 0.015	**0.007** 0.054
Status of employment			
SIMS-D score MARS-D score	0.175 0.218	0.031 0.048	0.269 0.072
Federal state of residence			
SIMS-D score MARS-D score	0.274 0.318	0.075 0.100	0.088 **0.013**
Primary source of information			
MARS-D score	0.226	0.051	0.08
Previous pregnancies			
SIMS-D score MARS-D score	0.009 0.031	0.001 0.001	0.881 0.631
Pregnancy complications			
SIMS-D score MARS-D score	0.083 0.077	0.007 0.006	0.311 0.349
Previous supplement intake aside from pregnancy and conception			
SIMS-D score MARS-D score	0.108 0.013	0.011 0.001	0.087 0.843
Experiences of complications during conception or pregnancy in the social environment, which were likely associated with a perceived lack of dietary supplementation			
SIMS-D score MARS-D score	0.089 0.111	0.008 0.012	0.159 0.08

MARS-D = German Medication Adherence Report Scale; SIMS-D = German Satisfaction with Information about Medicines Scale

The overall MARS-D score was 27.16 ± 3.06. Pregnant participants achieved a mean score of 27.47 ± 2.77, compared to 26.71 ± 3.41 among participants undergoing fertility treatment; this difference was not statistically significant (*p* = 0.054). Significant correlations were observed for pregnancy trimester (*p* = 0.017) and federal state of residence (*p* = 0.013). Participants in their first trimester achieved a mean score of 28.79 ± 1.50, and those in the third trimester had a mean of 27.14 ± 2.77. Those from Lower Saxony obtained a mean score of 26.96 ± 3.07, while participants from North Rhine-Westphalia showed a mean score of 27.98 ± 2.17, and the mean score of participants from other regions was 26.46 ± 4.31. The primary source of information showed no significant correlation with MARS-D results (gynecologist: 27.49 ± 2.66; internet: 27.17 ± 2.82; *p* = 0.08). Regarding under- or overdosages assessed by the MARS-D subscales, mean scores were 4.71 ± 0.66 and 4.69 ± 0.75, which corresponds to responses indicating “rarely” and “never.”

### Motivational aspects

A graphical overview of all statements and their mean responses can be found in the *Supplemental Figs. 3–6*.

Supplement intake was primarily motivated by protecting the unborn child (4.72 ± 0.55) and self-protection against deficiencies (4.22 ± 0.93). Trust in official sources (3.94 ± 1.00), fertility enhancement (3.55 ± 1.43), and doctor’s advice (3.44 ± 1.30) were also relevant. The most influential factor for non-adherence was a lack of awareness about the necessity of supplement intake (3.00 ± 1.41), followed by a low level of trust in the official information (2.29 ± 1.11) and deeming supplementation unnecessary (2.71 ± 1.11). A reason for indecisiveness was feeling overwhelmed by the oversupply of MMS (3.2 ± 1.64). Non-adherent participants indicated they might reconsider their decision after an in-depth educational conversation with their doctor (4.50 ± 0.71), if they read an article that explains the benefits of supplementation (4.00 ± 1.41), or if their health concerns were addressed more strongly (3.50 ± 0.71). Out of the undecided participants, 40.0% stated they would require additional education from their physician, while 20.0% preferred further information from the media. All non-adherent participants reported feeling strongly pressured to take supplements.

## Discussion

This study reveals a remarkably high adherence to micronutrient supplementation, particularly preconceptionally, exceeding rates reported in previous studies [25–29]. This encouraging finding likely reflects the inclusion of 40.6% of women undergoing fertility treatment, who are inherently in the preconceptional phase. The results highlight the positive role of fertility care in promoting supplement adherence. At the same time, the lower adherence rates reported in earlier studies may point to the need for broader outreach and education among women with unplanned pregnancies or those not engaged in fertility care [28, 29]. Strengthening population-wide educational strategies could help ensure that all women, regardless of their pregnancy planning status, receive adequate information and support to optimize preconception health.

Despite this high adherence, satisfaction with healthcare-provided information and awareness of recommended micronutrients, was relatively low. When considering the low satisfaction levels together with the limited knowledge of specific micronutrients, a discrepancy appears to emerge between behavioral adherence and informed decision-making. This might imply that supplement intake may be driven more by a general intention to comply than by a comprehensive understanding of the benefits and risks of micronutrient intake. This knowledge gap is especially concerning given the considerable variability in MMS formulations. As demonstrated in this and previous studies, many preparations differ significantly in composition and dosage, and participants reported taking on average two preparations concurrently [25, 27]. While it is encouraging that participants reported little to no variation in their intake behavior according to the MARS-D questionnaire, indicating consistent adherence to supplementation regimens, the absence of professional guidance may increase the risk of unintentional overdosing. Adverse effects of micronutrient overload, while rarely discussed and researched, remain a concern [1]. For instance, excessive oral iron intake during pregnancy is associated with hypertensive disorders, gestational diabetes, thrombotic events, and gastrointestinal side effects [6]. While 62.1% of the MMS consumed contained iron, which is consistent with another German study reporting an iron intake by 65% of participants [27], the WHO reports an anemia prevalence of only 15.4% among pregnant women in Germany [34]. Similarly, folic acid doses varied considerably, especially when taken as single-ingredient supplements. Some exceeded the upper daily limit of 1 mg, a level associated with increased risk of atopic airway diseases, insulin resistance, and potentially masking megaloblastic anemia [35]. A combination with vitamin B12 can mitigate the risk of megaloblastic anemia without posing significant risks of an overload in itself [36]. However, the necessity of a vitamin B12 supplementation is limited to individuals with specific medical histories or dietary restrictions, such as vegans or vegetarians [7]. Vitamin D intakes above 4000 I.U. per day over an extended period may result in hypercalcemia, particularly when combined with high calcium intake [37, 38]. DHA supplementation, though beneficial for those with low fatty fish or algae intake, can impair platelet function and immune responses at very high doses [7, 39, 40]. If fish and algae products are consumed regularly, it should also be considered that they can contain high levels of iodine and toxic heavy metals [7, 41, 42]. Excessive iodine intake can lead to maternal and fetal hyperthyroidism [9, 35]. Very high choline intake (above 3.5 g per day) is associated with a fishy body odor, vomiting, excessive sweating and salivation, hypotension, and liver toxicity [22, 43]. However, in our study, choline doses in preparations consistently remained below the recommended daily intake, and knowledge of this micronutrient was particularly poor. This suggests a likely underdosage and highlights the inadequacy of current educational strategies, which often emphasize a narrow set of micronutrients, most notably folic acid and iodine, while neglecting other important ones. Healthcare providers should shift from broad, generic advice to personalized guidance. The development of official guidelines and further training of healthcare professionals are therefore crucial to improve the quality of patient education [44, 45]. A combination of in-depth conversations with healthcare providers and trustworthy media sources may be most effective in ensuring comprehensive patient education and ensuring continuous access to high-quality information. In situations where consultation time is limited and given our findings indicating that it may be as effective as in-person consultations for disseminating information, the internet can play a crucial role in providing accurate guidance on supplementation. Further research should explore reliable educational resources.

Pregnant women, younger participants, and those with lower educational attainment reported higher satisfaction with the education they received. In contrast, older participants and those undergoing fertility treatment, who are subgroups that often face more complications, felt less well informed. Participants in the first trimester reported higher adherence than those in later trimesters, while participants from North Rhine-Westphalia had higher adherence scores compared to those from Lower Saxony and other regions. Although previous studies have associated higher educational levels, advanced age and primiparity with higher adherence rates [25–29, 46], these factors did not emerge as significant in our study. This study did not examine restrictive dietary regimens, planned pregnancy, or German language proficiency, yet these factors may also be linked to higher adherence rates [25, 27–29]. This emphasizes the importance of assessing individual sociodemographic factors to provide personalized, rather than generic, recommendations regarding supplement intake.

Regarding motivational aspects, protecting the unborn child and self-protection against micronutrient deficiency emerged as the strongest drivers for supplement intake. In contrast, a lack of awareness of the necessity of supplement intake was the most significant barrier to adherence, while undecided participants reported that being overwhelmed by the oversupply of available preparations was the greatest barrier. Both undecided and non-adherent participants expressed a need for more information from both gynecologists and media sources to reconsider the intake, highlighting the need for broader and easily understandable education.

To the best of our knowledge, this is the first prospective study in Germany to simultaneously examine adherence, perceived quality of education, knowledge, and motivational factors related to supplement intake during conception and pregnancy. The inclusion of both women seeking fertility treatment and pregnant women provides new insights, particularly into the preconceptional phase. The high response (38.9%) and completion rates (68.3%) enhance data reliability, and the use of two validated questionnaires strengthens the statistical power.

The study focused on selected key micronutrients and did not account for overall dietary intake, which is crucial in clinical contexts. Despite nationwide dissemination, most participants were from Lower Saxony and North Rhine-Westphalia and had a high level of education, resulting in a selection bias. The survey was only available in German, which may have excluded non-native speakers. Excluding incomplete responses improved data consistency but likely underrepresented less adherent or less educated individuals. The voluntary nature of participation may also have led to an underrepresentation of women skeptical of medical recommendations. Since the data are based on subjective perceptions, false findings cannot be ruled out, which is evident in the lower percentages of folic acid awareness after information provision. The validated SIMS-D questionnaire assesses participants’ subjective satisfaction with the information provided and does not objectively assess their actual knowledge. Participants’ level of understanding can therefore only be inferred in combination with other measures, such as the number of known recommended micronutrients, which are not included in any validated instrument.

To conclude, while adherence to supplementation recommendations is encouragingly high, substantial gaps in knowledge and education remain. Interventions combining personalized consultations with widely available, reliable media resources could help narrow down the knowledge gaps. A thorough assessment of individual needs and more specific supplement recommendations based on official guidelines could help healthcare providers prevent both under- and overdosage, ultimately contributing to better maternal and fetal outcomes.

## Supplementary Information

Below is the link to the electronic supplementary material.

Supplementary file1 (DOCX 56 KB)

Supplementary file2 (DOCX 24 KB)

Supplementary file3 (DOCX 1065 KB)

## Data Availability

The raw data supporting the conclusions of this article will be made available by the authors upon request, without undue reservation.
