# Supplementary material for: Adherence to vitamin and dietary supplement intake in fertility and pregnancy care: insights into knowledge, information satisfaction, and formulation variability
Source: Arch Gynecol Obstet. 2026 Jan 7;313(1):17. doi: 10.1007/s00404-025-08288-w (PMC12779681; doi:10.1007/s00404-025-08288-w)
Supplement: Supplementary file 1 — Supplementary file1 (DOCX 56 KB) [file 404_2025_8288_MOESM1_ESM.docx]

### **Adherence to Vitamin and Dietary Supplement Intake in Fertility and Pregnancy Care: Insights into Knowledge, Information Satisfaction, and Formulation Variability**

### Nele-Juliana Breuste^1^; Cordula Schippert^1^; Frauke von Versen-Höynck^1*^

### ^1^Hannover Medical School Department of Obstetrics and Gynecology, Hannover, Lower Saxony, Germany

*Corresponding author:
Prof. Dr. med. Frauke von Versen-Höynck, MD, MSc

E-Mail: vonversen-hoeynck.frauke@mh-hannover.de

**Supplemental Figures**

**Supplemental Fig. 1** Total SIMS-D score results categorized by reproductive status.

**Supplemental Fig. 2** Total MARS-D score results categorized by reproductive status. Dots (◯) represent mild outliers (1.5 – 3 × IQR) and stars (★) represent extreme outliers (>3 × IQR).

**Supplemental Fig. 3** Reasons for current or planned intake of vitamins and dietary supplements during pregnancy or conception. The given values are mean ratings and their SD from 1 (“totally disagree”) to 5 (“totally agree”) of the 5-point Likert scale.

**Supplemental Fig. 4** Reasons for not taking or for indecisiveness about the intake of vitamins and dietary supplements during pregnancy or conception. The given values are mean ratings and their SD from 1 (“totally disagree”) to 5 (“totally agree”) of the 5-point Likert scale.

**Supplemental Fig. 5** Factors that may influence a reconsideration of the decision to take vitamins and dietary supplements during pregnancy or conception of participants who denied the intake. The given values are mean ratings and their SD from 1 (“totally disagree”) to 5 (“totally agree”) of the 5-point Likert scale.

**Supplemental Fig. 6** Frequencies of factors that could influence a reconsideration of the decision to take vitamins and dietary supplements during pregnancy or conception of undecided participants.
