# Supplementary material for: Adherence to vitamin and dietary supplement intake in fertility and pregnancy care: insights into knowledge, information satisfaction, and formulation variability
Source: Arch Gynecol Obstet. 2026 Jan 7;313(1):17. doi: 10.1007/s00404-025-08288-w (PMC12779681; doi:10.1007/s00404-025-08288-w)
Supplement: Supplementary file 2 — Supplementary file2 (DOCX 24 KB) [file 404_2025_8288_MOESM2_ESM.docx]

### **Adherence to Vitamin and Dietary Supplement Intake in Fertility and Pregnancy Care: Insights into Knowledge, Information Satisfaction, and Formulation Variability**

### Nele-Juliana Breuste^1^; Cordula Schippert^1^; Frauke von Versen-Höynck^1*^

### ^1^Hannover Medical School Department of Obstetrics and Gynecology, Hannover, Lower Saxony, Germany

*Corresponding author:
Prof. Dr. med. Frauke von Versen-Höynck, MD, MSc

E-Mail: vonversen-hoeynck.frauke@mh-hannover.de

**Supplemental Tables**

**Supplemental Table 1** Detailed list of participants’ responses by federal state of residence.

| Federal state of residence | Participants | |
| --- | --- | --- |
|  | n = 254 | % |
| Baden-Württemberg | 3 | 1.2 |
| Bavaria | 3 | 1.2 |
| Berlin | 3 | 1.2 |
| Brandenburg | 0 | 0.0 |
| Bremen | 1 | 0.4 |
| Hamburg | 1 | 0.4 |
| Hesse | 3 | 1.2 |
| Mecklenburg-Western Pomerania | 0 | 0.0 |
| Lower Saxony | 164 | 64.6 |
| North Rhine-Westphalia | 64 | 25.2 |
| Rhineland-Palatinate | 5 | 2.0 |
| Saarland | 1 | 0.4 |
| Saxony | 1 | 0.4 |
| Saxony-Anhalt | 1 | 0.4 |
| Schleswig-Holstein | 2 | 0.8 |
| Thuringia | 0 | 0.0 |
| I don’t live in Germany. | 2 | 0.8 |

**Supplemental Table 2** Detailed list of participants’ primary source of information

| Primary source of information | Participants | |
| --- | --- | --- |
|  | n = 254 | % |
| Gynecologist | 96 | 37.8 |
| Pharmacy | 5 | 2.0 |
| Internet | 86 | 33.9 |
| Magazines/Brochures | 4 | 1.6 |
| TV | 3 | 1.2 |
| Friend/family/partner | 15 | 5.9 |
| School/university | 14 | 5.5 |
| Other, including:  Midwife  Books  Other medical specialties/clinics  Social media | 29 | 11.4 |
| I did not receive any information. | 2 | 0.8 |

**Supplemental Table 3** Micronutrient inclusion in 29 commercially available multiple micronutrient supplements (MMS)

| Micronutrient | Frequency of presence | |
| --- | --- | --- |
|  | n = 29 | % |
| Folic acid | 29 | 100.0 |
| Iodine | 24 | 82.8 |
| DHA | 12 | 41.4 |
| Iron | 18 | 62.1 |
| Vitamin B12 | 27 | 93.1 |
| Vitamin D | 24 | 82.8 |
| Choline | 7 | 24.1 |

DHA = Docosahexaenoic acid

**Supplemental Table 4** Number of participants reporting consumption of single-ingredient supplements of the recommended micronutrients

| Micronutrient | Participants | |
| --- | --- | --- |
|  | n = 254 | % |
| Folic acid | 25 | 10.5 |
| Iodine | 8 | 3.4 |
| DHA | 19 | 7.5 |
| Iron | 34 | 13.4 |
| Vitamin B12 | 18 | 7.1 |
| Vitamin D | 59 | 23.2 |
| Choline | 6 | 2.4 |

DHA = Docosahexaenoic acid
