# Supplementary material for: Adherence to vitamin and dietary supplement intake in fertility and pregnancy care: insights into knowledge, information satisfaction, and formulation variability
Source: Arch Gynecol Obstet. 2026 Jan 7;313(1):17. doi: 10.1007/s00404-025-08288-w (PMC12779681; doi:10.1007/s00404-025-08288-w)

### **Adherence to Vitamin and Dietary Supplement Intake in Fertility and Pregnancy Care: Insights into Knowledge, Information Satisfaction, and Formulation Variability**

### Nele-Juliana Breuste^1^; Cordula Schippert^1^; Frauke von Versen-Höynck^1*^

### ^1^Hannover Medical School Department of Obstetrics and Gynecology, Hannover, Lower Saxony, Germany

*Corresponding author:
Prof. Dr. med. Frauke von Versen-Höynck, MD, MSc

E-Mail: vonversen-hoeynck.frauke@mh-hannover.de

**Consent for participation in the study "Adherence to Vitamin and Dietary Supplement Intake in Pregnancy and Fertility Care: Insights into Knowledge, Information Satisfaction, and Formulation Variability"**

Dear Patient,

Thank you for your interest in participating in our study.
Since you are currently visiting a fertility center either for the first time or or are in gynecological care for your pregnancy, we would like to invite you to participate in this scientific survey. The aim of this study is to determine the level of knowledge and the intake rate of vitamins and dietary supplements in the context of conception and pregnancy. We hope to gather information that will enable us to offer better education and guidance to our patients. Before the survey begins, we ask you to read the detailed subject information with information on data protection. (Download Subject Information)
The data of the survey are confidential and are stored anonymously without any possibility of drawing conclusions about your person.
Demographic information such as age does not allow a clear conclusion about my person.
By participating in the study, I agree that my information may be collected and processed anonymously, without drawing conclusions about my individual person. The consent to the collection and processing of the data is irrevocable, as no participant-related deletion can be carried out due to the anonymized form of the survey.
After completion of the study, the data will be stored on MHH servers for 10 years and then irretrievably deleted.
I have read and understood the subject information. I had the opportunity to ask questions by phone or email.
All questions were answered satisfactorily.
I have had enough time to decide to participate in this study and I know that participation in this study is voluntary.

Hannover Medical School Department of Obstetrics and Gynecology
Molecular Perinatology and Reproductive Medicine Group, OE 6410
Prof. Dr. med. Frauke von Versen-Höynck
Phone: 0511 532-6080
vonversen-hoeynck.frauke@mh-hannover.de

After you give your consent by clicking on the first box, clicking on "Continue" will take you to our study.

**Please note that you can only participate in the survey if you have clicked the consent box below.**

- I agree that my data will be processed in accordance with the information listed here.
- I don't want to participate.

*🡪 If option 2 is selected: Interview will end*

*"We would like to thank you for your interest in our study and for looking at the information about it. Unfortunately, we were unable to persuade you to participate in the study.*

*However, if you change your mind and decide to participate in the study, the questionnaire is still available via the same link. You can use it to start over.*

*We would be happy to welcome you as a participant after all."*

**1. Are you pregnant or are you currently trying to conceive?**

Please select the appropriate option.

- I'm pregnant.
- I’m trying to conceive.
- I am neither pregnant, nor am I trying to conceive.

*🡪 if option 2 is selected: hide questions 6 & 7*

*🡪 if alternative option is selected (3): Interview is terminated as the inclusion criterion is to be pregnant or trying to conceive*

**2. How old are you?**

Please select the appropriate option.

- 17 or younger
- 18-20
- 21-29
- 30-39
- 40-49
- 50-59
- 60 or older

*🡪 if option 1 is selected, the interview will be terminated as the inclusion criterion to be at least 18 years old*

**3. In which federal state do you live?**

Please select the appropriate option.

- Baden-Württemberg
- Bavaria
- Berlin
- Brandenburg
- Bremen
- Hamburg
- Hesse
- Mecklenburg-Western Pomerania
- Lower Saxony
- North Rhine-Westphalia
- Rhineland-Palatinate
- Saarland
- Saxony
- Saxony-Anhalt
- Schleswig-Holstein
- Thuringia
- I don't live in Germany.

**4. What is the highest level of education you have completed?**

Please select the appropriate option.

- Left school without a diploma
- Completion of secondary school (9th grade, “Hauptschulabschluss”)
- Completion of intermediate school (10th grade, “Realschulabschluss”)
- Vocational Higher Education Entrance Qualification (“Fachabitur”)
- Completion of highschool (general university entrance qualification, “Abitur”)
- Completed vocational training
- University of applied sciences degree (Fachhochschulabschluss)
- University Degree
- Higher academic qualification (doctorate/habilitation)

**5. What is your current state of employment?**

Please select the appropriate option.

- Employed full-time
- Employed part-time
- Seeking work
- Independently
- On parental leave
- On leave/unable to work
- At school/university/training
- none of the options above

**6. In which trimester of pregnancy are you in?**

Please select the appropriate option.

- 1st trimester (1st-3rd month of pregnancy)
- 2nd trimester (4th-6th month of pregnancy)
- 3rd trimester (7th-9th month of pregnancy)

| **7. Have there been any complications during your pregnancy so far?**  Please select the appropriate option.   - Yes, the following complication(s): ___ - No.   **8. Have you been pregnant in the past?**  Please select the appropriate option.   - Yes. - No.   *🡪 if option 2 is selected: skip to question 11*  **9. How often have you been pregnant?**  Please enter the quantity.  Number of previous pregnancies: ___  **10. Please indicate how the previous pregnancies went.**   \| Number of live births: ___ \|  \| \| --- \| --- \| \| Number of miscarriages: ___ \|  \| \| Number of abortions: ___ \| \| \| Number of ectopic pregnancies: ___ \| \|   **11. Have you already had fertility treatments?**  Please select the appropriate option(s).   - Sexual intercourse at the optimal time - Intrauterine Insemination - Artificial insemination (IVF or ICSI) - Cryo-embryo transfer - Other:___ - None   **12. Where did you primarily obtain your information about taking vitamins and dietary supplements for conception and during pregnancy?**  Please select the most appropriate option.   - Gynecologist - Pharmacy - Internet - Magazines/Brochures - TV - friend/family/partner - School/university - Other:___ - I didn't receive any information. |
| --- | --- | --- | --- | --- | --- | --- | --- | --- |

**13. Which vitamins and micronutrients were you already aware of BEFORE receiving further information (through a doctor's consultation, through the Internet/TV/magazines, ...) as being necessary and recommended for conception and during pregnancy?**

Please tick the appropriate option(s).

- Folic acid
- Iodine
- Iron
- Vitamin B12
- Vitamin D
- Choline
- I didn't know about any of the vitamins or substances mentioned.

**14. Which vitamins and micronutrients were you already aware of AFTER receiving further information (through a doctor's consultation, through the Internet/TV/magazines, ...) as being necessary and recommended for conception and during pregnancy?**

Please tick the appropriate option(s).

- Folic acid
- Iodine
- Iron
- Vitamin B12
- Vitamin D
- Choline
- I did not receive any information and do not know about any of the vitamins or substances mentioned.

**In the following question, we ask you to assess your knowledge of vitamins and dietary supplements for conception and during pregnancy.**
"Medication" refers to **vitamins and dietary supplements.**Think in particular of the substances folic acid and iodine.

If no informative conversation has taken place yet, please select "No information received".

**15. SIMS-D Questionnaire**

The questionnaire is not displayed due to copyright restrictions. For access to the questionnaire, please contact the original author.

© Rob Horne. SIMS-D translation by the Department of General Practice and Health Services Research and Department of Internal Medicine VI, Clinical Pharmacology and Pharmacoepidemiology at Heidelberg University Hospital.

In the following question, we ask you to assess your **intake behavior**. Here, too, the term "medication" refers to **vitamins and dietary supplements for conception and during pregnancy.**

**16. MARS-D questionnaire**

The questionnaire is not displayed due to copyright restrictions. For access to the questionnaire, please contact the original author.

© Rob Horne. MARS-D translation by the Department of General Practice and Health Services Research and Department of Internal Medicine VI, Clinical Pharmacology and Pharmacoepidemiology of Heidelberg University Hospital.

**17. Are you currently taking vitamins and supplements for before and during pregnancy?**

- Yes.
- No.

*🡪 if option 2 is ticked: jump to question 22*

**18. Since when have you been taking vitamins and dietary supplements for before and during pregnancy?**

Please select the appropriate option.

- Since before conception or as part of my efforts to conceive
- After conception, since the 1st trimester of pregnancy (1st – 3rd month of pregnancy)
- After conception, since the 2nd trimester of pregnancy (4th – 6th month of pregnancy)
- After conception, since the 3rd trimester of pregnancy (7th – 9th month of pregnancy)
- I don't know.

**19. Please state how the following statements apply to you.**

**I take vitamins and dietary supplements for before and during pregnancy because...**

|  | Totally agree | Agree | I neither agree nor disagree | Disagree | Strongly disagree |
| --- | --- | --- | --- | --- | --- |
| I want to protect myself from a micronutrient deficiency. |  |  |  |  |  |
| I want to protect my child from a micronutrient deficiency. |  |  |  |  |  |
| a relative/friend asked me to do so. |  |  |  |  |  |
| my doctor advised to do so. |  |  |  |  |  |
| my pharmacist advised to do so. |  |  |  |  |  |
| a friend also takes them. |  |  |  |  |  |
| I trust the current information on the subject. |  |  |  |  |  |
| I see it as an opportunity to increase my fertility. |  |  |  |  |  |

**20. Other reasons: ___**

**21. What preparation(s) are you currently taking?**

Please select the appropriate option(s). If you are not taking a multiple micronutrient supplement (MMS), but individual micronutrients, please indicate the dose below if possible.

- A total of 48 German and commercially available MMS were listed as answer options; however, they are not displayed due to copyright reasons and to avoid potential conflicts of interest. The original list may be obtained upon request.
- Folic acid: ___ μg
- Iodine: ___ μg
- DHA (docosahexaenoic acid): ____ mg
- Iron: _____ mg
- Vitamin B12: ____ μg
- Vitamin D: ____ I.U.
- Choline: ____ mg
- Other MMS: ____
- I don't know.

*🡪 for all options: Jump to question 33*

**22. Do you plan on taking vitamins and dietary supplements for before and during pregnancy?**

Please select the appropriate option.

- Yes.
- No.
- I'm still undecided.

*🡪 if option 1 is selected: Skip to question 31*

*🡪 if option 3 is selected: skip to question 28*

**23. Please state how the following statements apply to you.**

**I do not take vitamins and dietary supplements for before and during pregnancy because...**

|  | Totally agree | Agree | I neither agree nor disagree | Disagree | Strongly disagree |
| --- | --- | --- | --- | --- | --- |
| I think the preparations are too expensive. |  |  |  |  |  |
| I did not know that the intake was necessary. |  |  |  |  |  |
| I do not think the intake is necessary. |  |  |  |  |  |
| I see dietary supplements as a health risk for myself. |  |  |  |  |  |
| I see dietary supplements as a health risk for my child. |  |  |  |  |  |
| A relative/friend advised against it. |  |  |  |  |  |
| my gynecologist advised against it. |  |  |  |  |  |
| I have a lack of trust in the current information on the subject. |  |  |  |  |  |
| I am not allowed to take any preparations for health reasons. |  |  |  |  |  |

**24. Other reasons: ____**

**25. Please state how the following statements apply to you.**

**I would reconsider my decision regarding the intake of vitamins and dietary supplements for conception and pregnancy if...**

|  | Totally agree | Agree | I neither agree nor disagree | Disagree | Strongly disagree |
| --- | --- | --- | --- | --- | --- |
| my doctor had an in-depth educational conversation with me. |  |  |  |  |  |
| I read an article that explains the benefits of supplementation. |  |  |  |  |  |
| a friend also took them. |  |  |  |  |  |
| the preparations became cheaper. |  |  |  |  |  |
| my health concerns were addressed more strongly. |  |  |  |  |  |

**26. Other factors: ___**

**27. How much pressure do you feel regarding the intake of vitamins and dietary supplements for conception or during pregnancy?**

Please select the appropriate option.


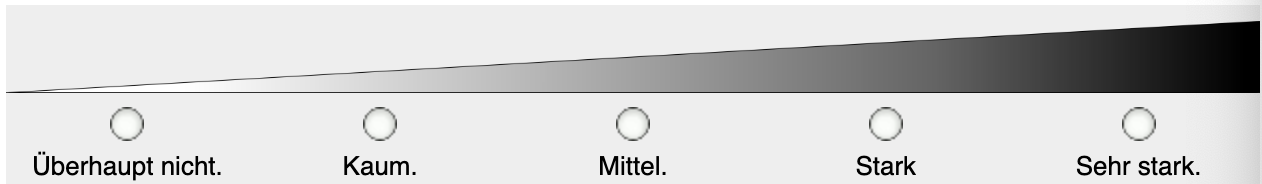


**Not at all Slight Moderate Strong Very strong**

*🡪 for all options: Jump to question 33*

**28. Please state how the following statements apply to you.**

**I am undecided whether I will take vitamins and dietary supplements for before and during pregnancy because...**

|  | Totally agree | Agree | I neither agree nor disagree | Disagree | Strongly disagree |
| --- | --- | --- | --- | --- | --- |
| I think the preparations are too expensive. |  |  |  |  |  |
| I do not think the intake is necessary. |  |  |  |  |  |
| I see dietary supplements as a health risk for myself. |  |  |  |  |  |
| I see dietary supplements as a health risk for my child. |  |  |  |  |  |
| a relative/friend advised against it. |  |  |  |  |  |
| my doctor advised against it. |  |  |  |  |  |
| I have a lack of trust in the current information on the subject. |  |  |  |  |  |
| I am not allowed to take any preparations for health reasons. |  |  |  |  |  |
| I did not want to take any, but a friend is taking them. |  |  |  |  |  |
| I did not want to take any, but my doctor urgently advised to do so. |  |  |  |  |  |
| I did not want to take any, but my pharmacist urgently advised to do so. |  |  |  |  |  |
| I am overwhelmed by the oversupply of preparations. |  |  |  |  |  |

**29. Other reasons:**

**30. In order to decide on the intake of vitamins and dietary supplements for pregnancy and concpetion...**

Please select the most appropriate option.

- I would need more information from the media.
- I would need more information from my family doctor.
- I would need more information from my gynecologist.
- I would need more information from my fertility doctor.
- further education would not help me either.

🡪 *for all options: Jump to question 33*

**31. Please state how the following statements apply to you.
In the future, I plan on taking vitamins and dietary supplements for before and during pregnancy because...**

|  | Totally agree | Agree | I neither agree nor disagree | Disagree | Strongly disagree |
| --- | --- | --- | --- | --- | --- |
| I want to protect myself from a micronutrient deficiency. |  |  |  |  |  |
| I want to protect my child from a micronutrient deficiency. |  |  |  |  |  |
| a relative/friend asked me to do so. |  |  |  |  |  |
| my doctor advised to do so. |  |  |  |  |  |
| my pharmacist advised to do so. |  |  |  |  |  |
| a friend also takes them |  |  |  |  |  |
| I see it as an opportunity to increase my fertility. |  |  |  |  |  |

**32. Other reasons:**

**33. Have you already taken vitamins or dietary supplements aside from pregnancy or conception?**

Please select the appropriate option.

- Yes.
- No.

**34. Have you ever had experiences of complications during conception or pregnancy in the social environment, which were likely associated with a perceived lack of dietary supplementation?**

Please select the appropriate option.

- Yes.
- No.

*“Thank you for participating in our study. Your answers have been saved; you can close the window.”*


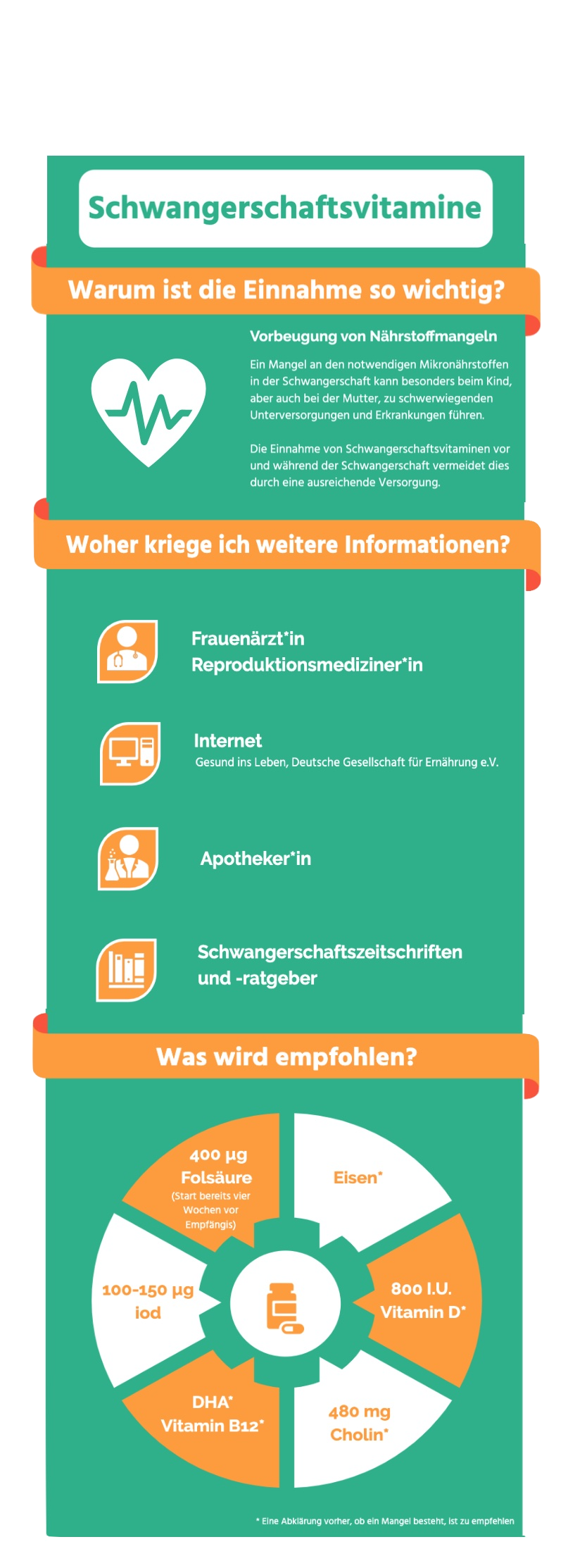

Supplement: Supplementary file 3 — Supplementary file3 (DOCX 1065 KB) [file 404_2025_8288_MOESM3_ESM.docx]
